# Functionalized selenium nanoparticles for targeted siRNA delivery silence Derlin1 and promote antitumor efficacy against cervical cancer

**DOI:** 10.1080/10717544.2019.1667452

**Published:** 2019-12-12

**Authors:** Yu Xia, Guoyi Tang, Changbing Wang, Jiayu Zhong, Yi Chen, Liang Hua, Yinghua Li, Hongsheng Liu, Bing Zhu

**Affiliations:** a Central Laboratory, Guangzhou Institute of Pediatrics, Guangzhou Women and Children’s Medical Center, Guangzhou Medical University, Guangzhou, China;; b Guangzhou First People's Hospital, School of Medicine, South China University of Technology, Guangzhou, China;; c Department of Obstetrics Gynecology, Guangzhou Women and Children’s Medical Center, Guangzhou Medical University, Guangzhou, China;; d Department of Radiology, Guangzhou Women and Children’s Medical Center, Guangzhou Medical University, Guangzhou, China

**Keywords:** Cervical cancer, tumor targeting, gene therapy, siRNA delivery, apoptosis

## Abstract

Small interfering RNA (siRNA) exhibits great potential as a novel therapeutic option due to its highly sequence-specific ability to silence genes. However, efficient and safe delivery carriers are required for developing novel therapeutic paradigms. Thus, the successful development of efficient delivery platforms for siRNA is a crucial issue for the development of siRNA-based drugs in cancer treatments. In this study, biocompatible selenium nanoparticles (SeNPs) were loaded with RGDfC peptide to fabricate tumor-targeting gene delivery vehicle RGDfC-SeNPs. Subsequently, RGDfC-SeNPs were loaded with Derlin1-siRNA to fabricate RGDfC-Se@siRNA, which are functionalized selenium nanoparticles. RGDfC-Se@siRNA showed greater uptake in HeLa cervical cancer cells in comparison with that in human umbilical vein endothelial cells (HUVECs), verifying the RGDfC-mediated specific uptake of RGDfC-Se@siRNA. RGDfC-Se@siRNA was capable of entering HeLa cells via clathrin-associated endocytosis, and showed faster siRNA release in a cancer cell microenvironment in comparison with a normal physiological environment. qPCR and western blotting assays both indicated that RGDfC-Se@siRNA exhibited an obvious gene silencing efficacy in HeLa cells. RGDfC-Se@siRNA suppressed the invasion, migration and the proliferation of HeLa cells, and triggered HeLa cell apoptosis. Moreover, RGDfC-Se@siRNA induced the disruption of mitochondrial membrane potentials. Meanwhile, RGDfC-Se@siRNA enhanced the generation of reactive oxygen species (ROS) in HeLa cell, suggesting that mitochondrial dysfunction mediated by ROS might play a significant role in RGDfC-Se@siRNA-induced apoptosis. Interestingly, RGDfC-SeNPs@siRNA exhibited significant antitumor activity in a HeLa tumor-bearing mouse model. Additionally, RGDfC-SeNPs@siRNA is nontoxic to main organ of mouse. The above results indicate that RGDfC-Se@siRNA provides a promising potential for cervical cancer therapy.

## Introduction

Cervical cancer is one primary cause of gynecologic malignancy among women all over the world, and approximately 580,000 new cervical cancer patients are diagnosed every year, of which China accounts for more 10% of these new cervical cancer cases (Yang et al., 2017). Human papilloma virus (HPV) has been reported to be the main cause of cervical cancer, although some other factors may also be involved (Zhou et al., 2017). The current neoadjuvant radiotherapy and chemotherapy combination is satisfactory for early-stage cervical cancer patients (Zhang et al., 2016). However, the therapeutic effect for advanced-stage cervical cancer patients is still unsatisfactory (Lim et al., 2017). Moreover, because conventional chemotherapy is usually accompanied by severe systemic toxicity and chemoresistance, safe and effective therapeutic strategies against cervical cancer are urgently needed (Zhao et al., 2019). Gene silencing by short interfering RNA (siRNA) provides a promising prospect for cervical cancer therapy (Li et al., 2019a). Although viruses have been used as a gene delivery vehicle, the viral vehicle is prone to the risk of insertional mutagenesis and immunogenicity (Herma et al., 2019). Thus, nonviral vehicles hold enormous potential for cancer gene therapy because of their superior safety (Kim et al., 2018).

There has been wide use of selenium nanoparticles (SeNPs) in the field of anticancer drug/gene vehicles (Zheng et al., 2016). The trace element selenium (Se) can exhibit unique activity for preventing the occurrence of cancer, and it also reduces drug toxicity, regulates the function of the thyroid gland, and ensures proper functioning of immune system, thus playing a major role in combating disease (Zhou et al., 2016). Because of these advantages, SeNPs are superior to existing strategies including biogenetic (exosome, modularized extracellular vesicles, or extracellular vesicle-mimetic) and synthetic (inorganic nanoparticle and liposome) (Chen et al., 2015). Therefore, SeNPs have great potential as chemotherapeutic drugs/gene vehicles (Xia et al., 2019a). However, the lack of active targeting capability is still an issue that needs to be solved (Sun et al., 2014). In order to acquire active targeting capability, many molecules (e.g., arginylglycylaspartic acid (RGD) peptide, folate, and hyaluronic acid) have been linked to surfaces of nanoparticles to function as tumor-targeting moieties (Xia et al., 2017b, 2019b). Herein, in order to prepare an active targeting delivery vesicle, a positive charge peptide RGDfC was chosen for installation on the surfaces of SeNPs to prepare gene delivery vesicle RGDfC-SeNPs because the RGDfC peptide can specifically bind to α_v_β_3_ integrin, which is highly expressed in kinds of cancer cells, including HeLa cervical cancer cell (Zhang et al., 2017). In addition, positively charged RGDfC-SeNPs are beneficial for binding with siRNA through their electrostatic interaction (Li et al., 2018a).

Derlin1 is an endoplasmic reticulum membrane protein that is responsible for transporting unfolded or misfolded proteins from endoplasmic reticulum lumen to cytoplasm (Li et al., 2019b). Increasing evidence has shown that Derlin1 is overexpressed in most kind of cancer, including cervical cancer and the expression of Derlin1 is closely related to the occurrence and development of tumors. (Liang et al., 2014; Yang et al., 2018). Derlin1 knockdown inhibited cell migration in a bladder cancer cell model (Li et al., 2016). Derlin1 antibody inhibited tumor growth in a colon cancer mouse model (Oresic et al., 2009). Thus, Derlin1 has developed into a potential target for tumor gene-silencing therapy. Herein, RGDfC-SeNPs were loaded with Derlin1-siRNA to fabricate RGDfC-Se@siRNA, with the goal of silencing the Derlin1 gene. The *in vitro* and *in vivo* anticancer activity and mechanism of RGDfC-Se@siRNA were investigated in a cervical cancer tumor model with HeLa cells.

## Materials and methods

### Materials

Propidium, vitamin C (Vc), Sodium selenite (Na_2_SeO_3_), and DAPI were provided from Sigma (St. Louis, MO, USA). Fetal bovine serum (FBS) and Dulbecco’s modified Eagle’s medium (DMEM) was provided from Gibco. The antibody was provided from Cell Signaling Technology (MA, USA). siRNA was obtained from GenePharma Co., Ltd (Shanghai, China), and the sequence was as follows: Derlin1-siRNA (5′-GGGAGAGUCUGAACCUUAAUU-3′).

### Fabrication and characterizations of nanoparticle

Selenium nanoparticles (SeNPs) was fabricated according to previous studies (Li et al., 2017). In brief, 1 mM vitamin C (Vc) solution, 0.2 M Na_2_SeO_3_ solution, and 1.5 mg/mL RGDfC solution were freshly prepared. A solution was prepared that contained 4 mL vitamin C and 0.5 mL Na_2_SeO_3_, and gently stirred for 1.5 h to manufacture SeNPs. After that, 1 mL RGDfC was added to the SeNP solution to prepare RGDfC-SeNPs. The RGDfC-SeNP solution was stirred for 6 h and dialyzed for 4 h to acquire pure RGDfC-SeNPs. The morphologys of RGDfC-SeNPs were characterized via transmission electron microscopy (TEM). Elemental compositions of RGDfC-SeNPs were examined via energy dispersive spectroscopy (EDS). Fourier transform infrared spectroscopy (FTIR) was applied to characterize chemical structures of RGDfC-SeNPs. Zeta potentials and size distributions of RGDfC-SeNPs were observed with a Malvern Zetasizer.

The RGDfC-Se@siRNA complex was prepared by slowly dripping 100 nM Derlin1-siRNA into a solution of RGDfC-SeNPs for 40 min at 15 °C. The N/P ratio of RGDfC-Se@siRNA was 1/1, 2/1, 4/1, or 8/1, respectively. The concentrations of loaded siRNA were examined as previously described (de Almeida et al., 2017).

### Gel electrophoresis assay

RGDfC-Se@siRNA complexes with different N/P ratios were fabricated. RGDfC-Se@siRNA was subject to agarose gel electrophoresis (1%) for 12 min at 140 mV, and the gels were photographed with a gel imaging system. In order to determine if RGDfC-SeNPs could protect siRNA in serum, the electrophoretic migration experiment with RGDfC-Se@siRNA was carried out.

### Cell culture

Human umbilical vein endothelial cell (HUVEC) and HeLa human cervical cancer cell was provided from American Type Culture Collection (ATCC) and were cultivated in DMEM with 10% FBS in an incubator (80% humidity, Thermo Scientific) with 5% CO_2_ at 37 °C.

### Cellular uptake assay

To culture the cells, 2 mL HeLa cell suspensions (5 × 10^4^ cells/mL) were incubated in a 6-well plate overnight. Then, HeLa cell was exposed to RGDfC-Se@FAM-siRNA containing 100 nM FAM-siRNA. After that, HeLa cells were processed as previously described^22^ and photographed using a fluorescence microscope. The uptakes of RGDfC-Se@siRNA in HUVECs was analyzed via a similar method. Various uptake inhibitors were applied to study the cellular uptake mechanism of RGDfC-Se@siRNA. HeLa cells were processed as previously reported (Yin et al., 2015). The collected cells were examined via flow cytometry (Becton, Dickinson & Company, BD FACSAria II).

### siRNA release from nanoparticles

In order to examine released siRNA, RGDfC-Se@siRNA complex at the N/P rate of 8:1 was incubated in 2-[4-(2-hydroxyethyl)-1-piperazinyl]ethanesulfonic acid (HEPES) buffer (pH 7.4 and 5.4). The sample was removed from the incubator at a scheduled time, and siRNA concentrations were tested with a Spectromax Quickdrop spectrophotometer.

### Quantitative real-time PCR (qRT-PCR)

HeLa cells were incubated overnight to reach 80% confluences. HeLa cells were washed with PBS before transfection and then exposed to 100 nM RGDfC-Se@siNC (negative control) or RGDfC-Se@siRNA for 24 h. The previous medium was discarded and replaced with fresh one. HeLa cells were then incubated for 36 h. The untreated cell was set as the control group. TRIzol reagent was used to extract the total RNA from HeLa cells and a NanoDrop™ 1000 spectrophotometer was used to measure the RNA concentration; the operation was performed on ice. The data were analyzed by the StepOne™ PCR System using the 2^−△△CT^ method and primer sequence is shown in Table 1.

**Table 1.** The sequence primer used for quantitative real-time PCR.

Gene	Direction	Primers (5′–3′)
Derlin1	F	CGCTTTCAGATTTGGAGGCC
	R	GCCTCCCATCAAAAGCTCCT
GAPDH	F	GACTTCAACAGCGACACCCA
	R	CACCCTGTTGCTGTAGCCAAA

### Western blot assay

The protein level of HeLa cells was examined via western blotting. HeLa cells were incubated in a 6-cm dish to reach approximately 80% confluence. HeLa cells were exposed to RGDfC-Se@siNC or RGDfC-Se@siRNA at 100 nM of siRNA for 24 h and then collected for further analysis as previously reported (Zheng et al., 2014).

### *In vitro* wound healing analysis

Cell migrations were determined via wound healing assay. In brief, HeLa cell was incubated in 12-well plate, with each well containing 8 × 10^4^ cells, and the plate was then incubated overnight to reach 100% confluences. A 10-µL pipette tip was used to scratch the layer of cells, and residual cells were rinsed and added with DMEM (3% FBS). HeLa cells were treated with RGDfC-Se@siNC or RGDfC-Se@siRNA at 100 nM of siRNA for 12 h. Scratched monolayer cells were photographed (Leica DMi8 digital microscope) at 0 h and after further incubation for 12 h, respectively. The migration rate over a 12-h period was calculated by using following formula: cell motility (%) = (the average distances of wound at 0 h - the average distance of wound at 12 h/the average distance of wound at 0 h) ×100.

### *In vitro* trans-well assay

Invasion assays were carried out using a trans-well chamber (8 μm). HeLa cell was incubated in the serum-free medium overnight. Subsequently, the cells were washed and resuspend with serum-free medium. The cell suspension was added to upper chamber and the fresh complete medium was added to lower chamber. The HeLa cells were incubated with RGDfC-Se@siNC or RGDfC-Se@siRNA at 100 nM siRNA for 24 h. HeLa cell on upper filter was gently wiped. The invasive cell under lower membrane surface was stained using crystal violet (0.1%) for 10 min. The invading cells in five different views were photographed and counted via a microscope.

### MTT assay

3-(4,5-Dimethyl-2-thiazolyl)-2,5-diphenyl-2H-tetrazolium bromide (MTT) was applied to assess cellular toxicity of nanoparticles. Briefly, HeLa cell was incubated in 96-well plate overnight to reach 50% confluences. Subsequently, HeLa cells were co-incubated with RGDfC-Se@siNC or RGDfC-Se@siRNA (different siRNA concentration) for 48 h. Finally, HeLa cells were processed as previously reported (Mahajan et al., 2018).

### Flow cytometry assay

Flow cytometry (Becton, Dickinson & Company, BD FACSAria II) was applied to assess the cycle distributions and apoptosis of HeLa cells. Briefly, HeLa cells were co-incubated with RGDfC-Se@siNC or RGDfC-Se@siRNA at 100 nM of siRNA for 24 h. HeLa cell was rinsed and stained with PI in dark for 30 min. Finally, HeLa cell was examined by flow cytometry (Li et al., 2018b).

### Detection of mitochondrial membrane potential (*△Ψ*_m_)

Mitochondria depolarization was tested by JC-1 monomers to evaluate the status of △Ψ_m_ (Yuan et al., 2019). In Brief, the HeLa cells were exposed to RGDfC-Se@siNC or RGDfC-Se@siRNA containing 100 nM siRNA for 24 h. Subsequently, HeLa cell was washed and stained with JC-1 in dark for 20 min. Finally, the cells were collected for quantitative analysis by flow cytometry.

### The assessment of reactive oxygen species (ROS)

ROS in HeLa cells were detected as previously described (Garcia-Mazas et al., 2017). In brief, after HeLa cell was exposed to RGDfC-Se@siNC or RGDfC-Se@siRNA at 100 nM of siRNA dose for 24 h, HeLa cell was treated with 10 μM of DCFH-DA staining for 15 min. Subsequently, fluorescence images of cells were photographed via fluorescence microscope, and the fluorescence intensities of cells were detected with a fluorescence plate reader.

### Xenograft mouse model

BALB/c nude mice with six-week-old were fed in an SFP-grade animal center (day–night cycle) and applied to investigate anticancer activity of RGDfC-Se@siRNA *in vivo*. 2 × 10^6^ HeLa cells was injected into the abdomen of mouse subcutaneously. The mice were randomly assigned to three groups (*n* = 6) when tumor volumes reached approximately 100 mm^3^, Then, saline, RGDfC-Se@siNC, or RGDfC-Se@siRNA containing 0.2 mg/kg of the equivalent siRNA were intravenously injected to HeLa tumor-bearing mice by the tail vein once every other day for 21 days. The volumes of tumor were calculated with equation as follows: $\text{The}\;\text{volume}\;\text{of}\text{tumor}\;\left({\text{mm}}^{3}\right)\;=\frac{1}{2}\times \text{length}\times {\text{width}}^{2}.$  

Hematoxylin and eosin (H&E), caspase-3, pp53 and Bax staining was used for histologic examination. The sections were observed under an inverted fluorescence microscope (Leica DMi8). All animal experiments were approved by the Ethics Committee of Guangzhou Medical University.

### Statistical analysis

The data was expressed as mean ± standard deviations (SD). The statistical difference was analyzed via one-way analysis of variance (ANOVA). Differences with *p < .05 and **p < .01 were considered statistically significant.

## Results and discussion

### Synthesis and characterizations of RGDfC-SeNPs

**Figure 1.**
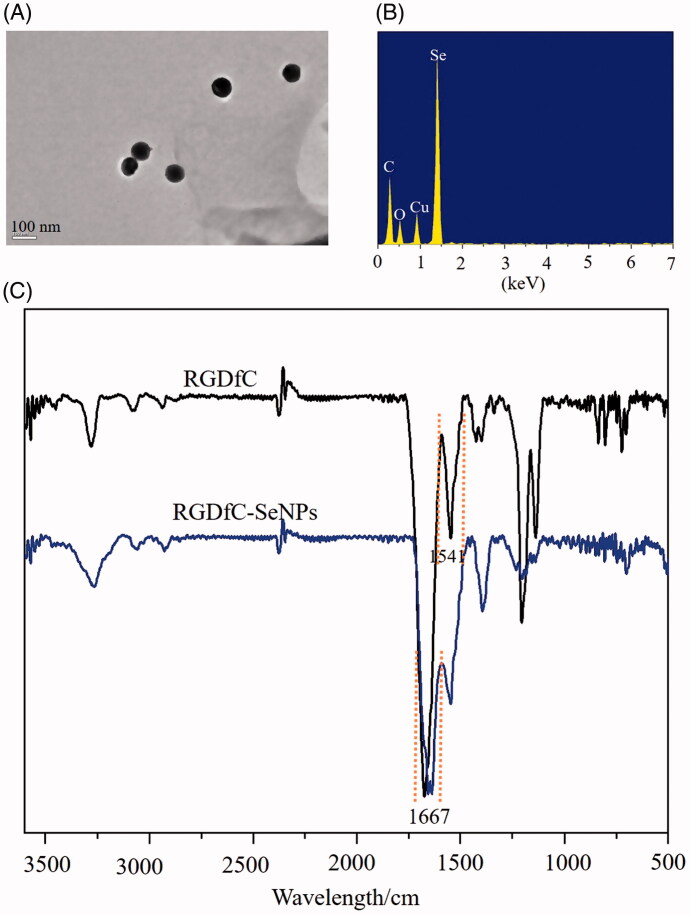
Characterizations of RGDfC-SeNPs nanoparticles. (A) TEM image of RGDfC-SeNPs. (B) EDS analysis of RGDfC-SeNPs. (C) FT-IR spectra of RGDfC peptide and RGDfC-SeNPs.

**Figure 2.**
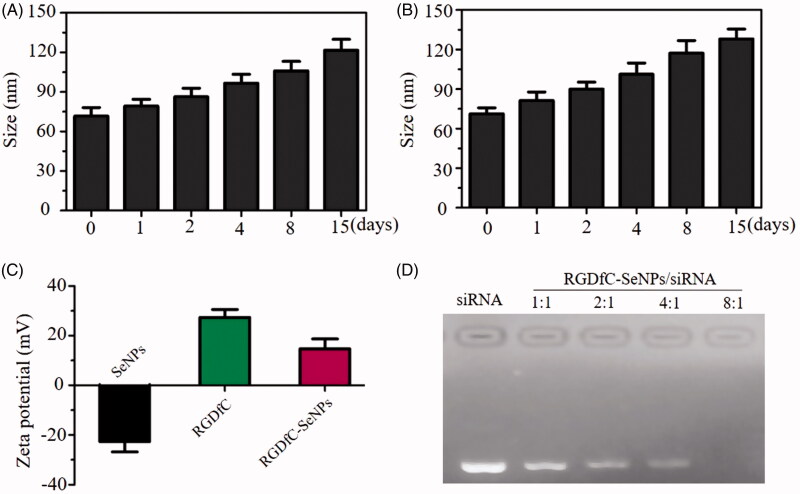
(A) Particle size observation of RGDfC-SeNPs in water. (B) Particle size observation of RGDfC-SeNPs in PBS. (C) Zeta potential of RGDfC, RGDfC-SeNPs, and SeNPs. (D) Gel electrophoresis retardation assay of RGDfC-Se@siRNA with different N:P rate from 1:1 to 8:1.

Tumor-targeting nanoparticle RGDfC-Se@siRNA was fabricated in this study. The tumor-targeting molecular cyclic peptide RGDfC was loaded on surfaces of selenium nanoparticles (SeNPs) to prepare siRNA carrier RGDfC-SeNPs. Subsequently, Derlin1-siRNA was linked with the RGDfC to fabricate tumor-targeting nanoparticles RGDfC-Se@siRNA. The morphology, particle size, and chemical structure of nanoparticles were characterized by various chemical methods. The morphology of RGDfC-SeNPs was characterized by TEM, which showed that RGDfC-SeNPs were composed of spherical particles approximately 75 nm in diameter (Figure 1(A)). Elemental analysis showed that an obvious signal of Se atoms from the SeNPs, and carbon and oxygen atom signals from RGDfC were also observed in spectrums of RGDfC-Se@siRNA (Figure 1(B)), suggesting that RGDfC was effectively loaded on the surfaces of SeNPs. Moreover, FTIR spectrum of RGDfC-SeNPs (Figure 1(C)) was similar to that of RGDfC, and the characteristic amide peak of RGDfC at 1667 and 1541 cm^−1^ were obviously observed in spectrums of RGDfC-SeNPs, suggesting the effective formation of RGDfC-SeNPs. As shown in Figure 2(A,B), the size distribution of RGDfC-SeNPs nanoparticles in water and phosphate buffered saline (PBS) indicated that RGDfC-SeNPs were stable with small size (<150 nm) during a 15-day observation period. These data showed that RGDfC-SeNPs had favorable stability in water and PBS. Seen from Figure 2(C), zeta potential of SeNPs increased from −22.6 mV to 14.7 mV after loading with RGDfC.

### Study on siRNA loading ability

The capability of RGDfC-SeNPs to load siRNA was assessed by agarose gel assay (Xia et al., 2017a). Figure 2(D) shows that RGDfC-SeNPs significantly bound to siRNA from N/P ratio of 1:1 to 8:1, and the migration of siRNA was retarded completely at the N/P ratio of 8:1. RGDfC-SeNPs were able to effectively bind to siRNA to hamper its degradation during the gel electrophoresis. The above result indicated that RGDfC-SeNPs exhibited great capability to protect siRNA from degradation.

### Selective uptake of RGDfC-Se@siRNA nanoparticles

**Figure 3.**
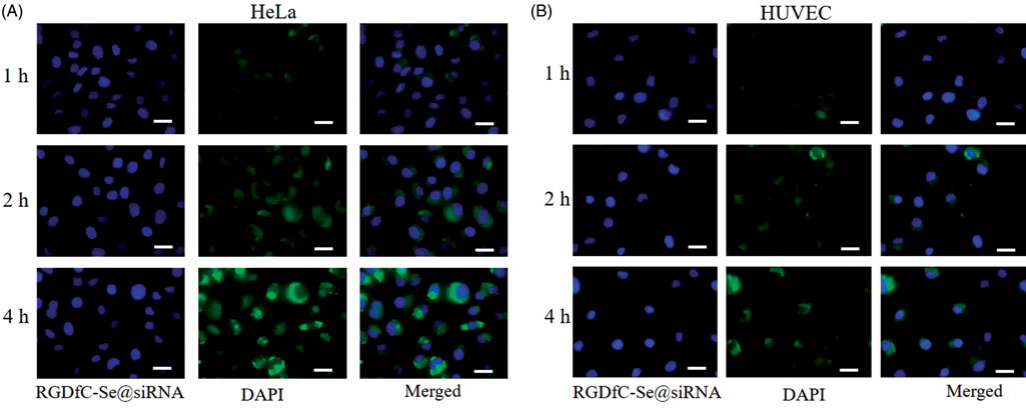
Cellular uptakes of RGDfC-Se@siRNA in (A) HeLa cells and (B) HUVECs was photographed via fluorescence microscope. Scale bar is 20 μm.

Drug delivery efficacy significantly depends on the cellular uptake of drugs. Effective uptake of drugs is a crucial issue for favorable treatment outcomes (Liang et al., 2018). Many studies have shown that RGD receptor α_v_β_3_ integrin is overexpressed in cancer cells (Oe et al., 2014). Therefore, the peptide RGDfC is used as a cancer-targeted moiety. The selective RGDfC-mediated uptake of RGDfC-Se@siRNA between HeLa cells and HUVECs was analyzed via fluorescence microscope. Seen from Figure 3, the uptake of RGDfC-Se@siRNA in HeLa cells was greater than that in HUVECs under the same conditions, verifying RGDfC-mediated specific uptake. Furthermore, the fluorescence intensities of HeLa cells exposed to RGDfC-Se@siRNA were gradually strengthened with increasing time, indicating that RGDfC-Se@siRNA internalized into HeLa cells through the time-dependent manner.

**Figure 4.**
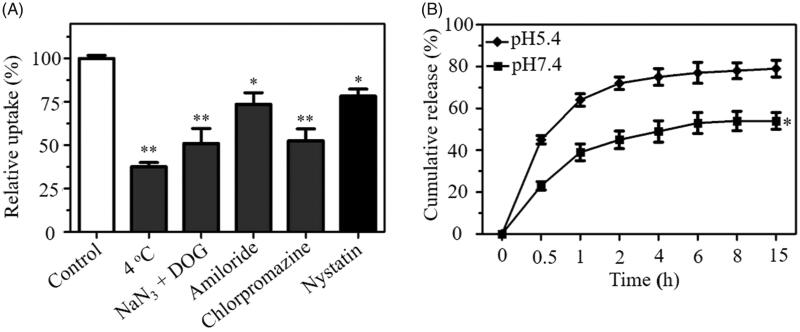
(A) Effects of temperature and endocytosis inhibitor on internalization of RGDfC-Se@siRNA in HeLa cells. *p < .05, **p < .01 vs control. (B) *In vitro* release analysis of siRNA from RGDfC-Se@siRNA in different pH value (pH 7.4 or 5.4). *p < .05 vs pH 5.4 group.

Endocytosis is an important mechanism for transporting nanoparticles across the cell membrane (Baranello et al., 2015). The uptake pathways of RGDfC-Se@siRNA were investigated by an uptake inhibition assay. Pretreatment with NaN_3_/DOG or incubation at 4 °C obviously reduced uptake of RGDfC-Se@siRNA in HeLa cells (Figure 4(A)), suggesting that RGDfC-Se@siRNA entered the cells via an energy-dependent endocytosis. The endocytosis mechanism of RGDfC-Se@siRNA was studied using different endocytosis inhibitors. Amiloride, chlorpromazine or nystatin was applied to suppress macropinocytosis, clathrin-associated endocytosis or caveolae-mediated endocytosis. As shown in Figure 4(A), after pretreatment with amiloride or nystatin, the uptake of RGDfC-Se@siRNA was reduced by 21.7% or 26.3%. Nevertheless, the pretreatment with chlorpromazine reduced the uptake of RGDfC-Se@siRNA by 46.3%, indicating that RGDfC-Se@siRNA entered cancer cells mainly by clathrin-associated endocytosis.

### *In vitro* release of siRNA

Different pH (pH 7.4 or 5.4) value was chosen to imitate the normal physiological environment or the cancer cell microenvironment (Mendes et al., 2019). Figure 4(B) showed that the release profile of siRNA from RGDfC-Se@siRNA nanoparticles indicated that there was a remarkable burst of siRNA release under both pH values during the initial 2 h. Interestingly, RGDfC-Se@siRNA showed a quicker release of siRNA at pH 5.4 during 15 h, with a release rate of approximately 79%. The release rate of siRNA at pH 7.4 was 54% at 15 h. The faster release of siRNA at pH 5.4 might be due to the increasing protonation of RGDfC-SeNPs at acidic conditions, which weakened electrostatic attraction between siRNA and RGDfC-SeNPs. Thus, acidic conditions facilitate release of siRNA from RGDfC-SeNPs, which is a favorable advantage for cancer treatment.

### RGDfC-Se@siRNA downregulates the gene expression of Derlin1

**Figure 5.**
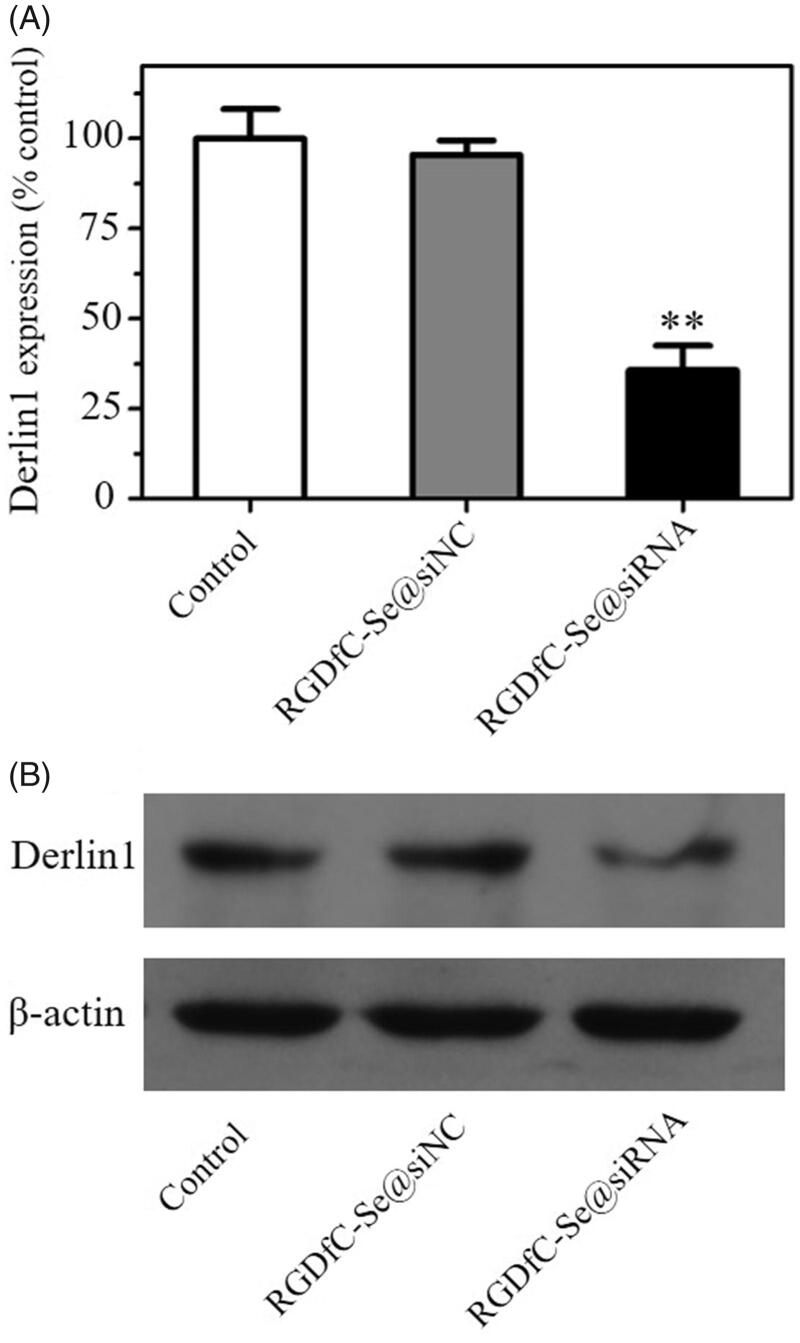
(A) The relative mRNA level of Derlin1 in HeLa cells treated with RGDfC-Se@siNC or RGDfC-Se@siRNA was analyzed by qRT-PCR. **p < .01 vs control. (B) The protein expression level of Derlin1 in HeLa cell.

RGDfC-SeNPs was used to carry Derlin1-siRNA to HeLa cells for silencing the expression of the Derlin1 gene. The mRNA level of Derlin1 in HeLa cells exposed to RGDfC-Se@siNC or RGDfC-Se@siRNA for 24 h was examined via qRT-PCR. Figure 5(A) shows that RGDfC-Se@siNC did not downregulate the expression of the Derlin1 gene. However, RGDfC-Se@siRNA obviously downregulated the Derlin1 mRNA levels in HeLa cells. The protein expression levels of Derlin1 in HeLa cells were analyzed using western blot technology. Seen from Figure 5(B), RGDfC-Se@siRNA observably inhibited the protein level of Derlin1 in HeLa cells. Nevertheless, RGDfC-Se@siNC exhibited an insignificant effect on the expression of Derlin1 protein, indicating that such an siRNA delivery carrier did not exhibit nonspecific gene silencing in HeLa cells. qRT-PCR and western blot results both indicated that RGDfC-Se@siRNA effectively silenced Derlin1 gene in HeLa cells.

### Effects of RGDfC-Se@siRNA on the invasion/migration of HeLa cells

**Figure 6.**
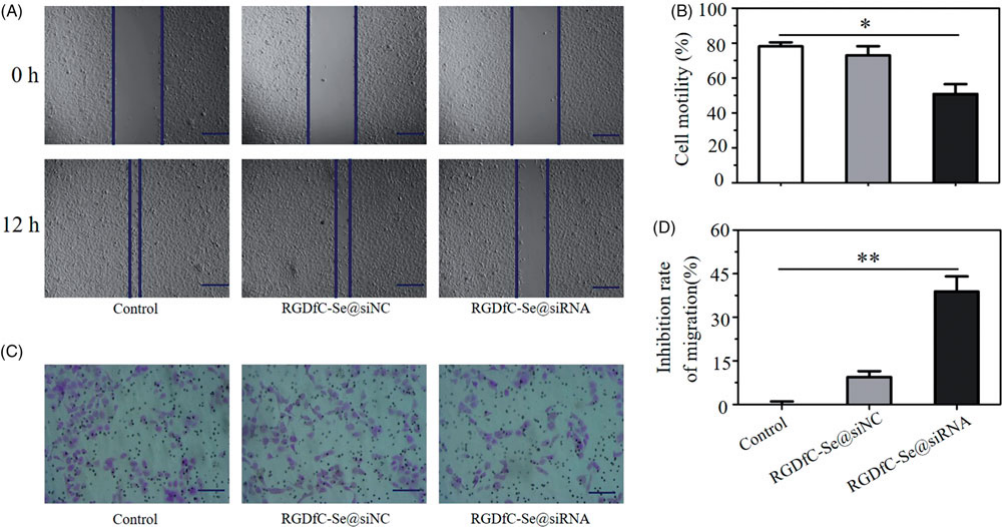
(A) The wound width of HeLa cells was photographed after 12 h of co-incubation with RGDfC-Se@siNC or RGDfC-Se@siRNA. Scale bar is 400 µm. (B) The quantitative analysis of cell motility in control, RGDfC-Se@siNC and RGDfC-Se@siRNA groups. (C) The effects of RGDfC-Se@siNC or RGDfC-Se@siRNA on the migration of HeLa cell. Scale bar is 200 µm. (D) The quantitative analysis of cell migration inhibition rate in control, RGDfC-Se@siNC or RGDfC-Se@siRNA groups. *p < .05, **p < .01 vs control.

Effective inhibition of tumor cell invasion/migration is considered as an important method for delaying the growth of tumor (Xia et al., 2018). The trans-well/wound healing assays were used to estimate whether RGDfC-Se@siRNA had the capability to suppress the invasion/migration of HeLa cells. Seen from Figure 6(A,C), RGDfC-Se@siRNA obviously inhibited the invasion/migration of HeLa cells. Moreover, RGDfC-Se@siRNA showed a greater capability to inhibit the invasion/migration of HeLa cells in comparison with RGDfC-Se@siNC (Figure 6(B,D)), which exerted an almost negligible effect on the invasion/migration of HeLa cells. The above result shows that RGDfC-Se@siRNA could inhibit the invasion/migration of HeLa cell through downregulating expression level of Derlin1 gene in HeLa cells.

### RGDfC-Se@siRNA inhibits the viability of HeLa cell

**Figure 7.**
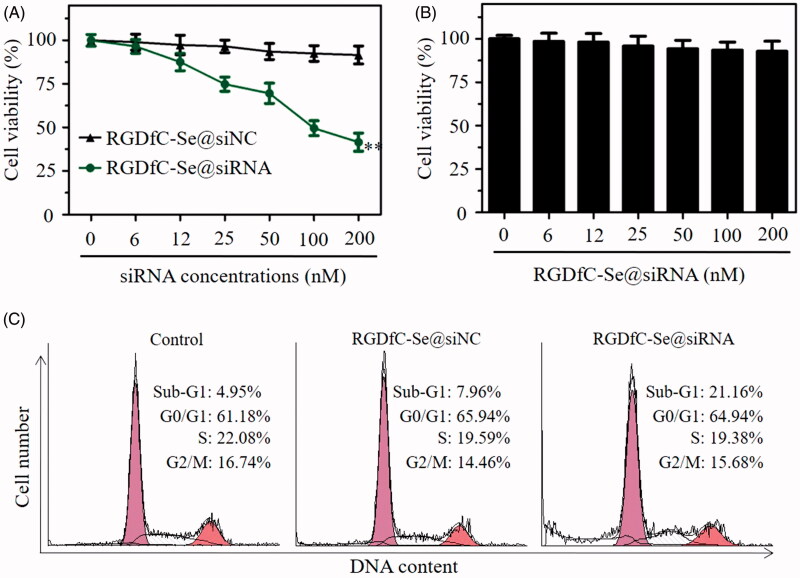
(A) The cytotoxicity of RGDfC-Se@siNC and RGDfC-Se@siRNA at various siRNA concentrations against HeLa cells. (B) The cytotoxicity of RGDfC-Se@siRNA against HUVECs. **p < .01 vs RGDfC-Se@siNC group. (C) Effect of RGDfC-Se@siNC or RGDfC-Se@siRNA on the cell cycle distributions and apoptosis in HeLa cells.

The MTT method was performed to investigate if RGDfC-Se@siRNA was capable of inhibiting the viability of HeLa cells. RGDfC-Se@siNC was used as a negative control. Figure 7(A) showed that the viability of HeLa cell was obviously suppressed, and the cell viability rate was 41.3% after 48 h of RGDfC-Se@siRNA treatment. In contrast, RGDfC-Se@siNC exhibited a negligible effect on the viability of HeLa cells. This result showed that RGDfC-Se@siRNA exhibited anticancer activity in HeLa cervical cancer cells by silencing the Derlin1 gene. Additionally, at the used dose, RGDfC-Se@siRNA exhibited slight toxicity against HUVECs (Figure 7(B)).

Flow cytometry was adopted to verify if RGDfC-Se@siRNA could trigger HeLa cell apoptosis. As shown in Figure 7(C), the sub-G1 peak reflected the apoptosis of cells, and the untreated cells were used as a control group. This result suggested that the sub-G1 apoptosis peak was more obvious (21.16%) in RGDfC-Se@siRNA group than that in RGDfC-Se@siNC group (7.96%) and control group (4.95%), indicating that RGDfC-Se@siRNA presented significant potential to trigger the apoptosis of HeLa cells. Nevertheless, no obvious cell cycle distribution difference was observed in the different groups. The above data showed that RGDfC-Se@siRNA could significantly trigger the apoptosis of HeLa cells.

### RGDfC-Se@siRNA induces mitochondrial dysfunction through ROS overproduction

**Figure 8.**
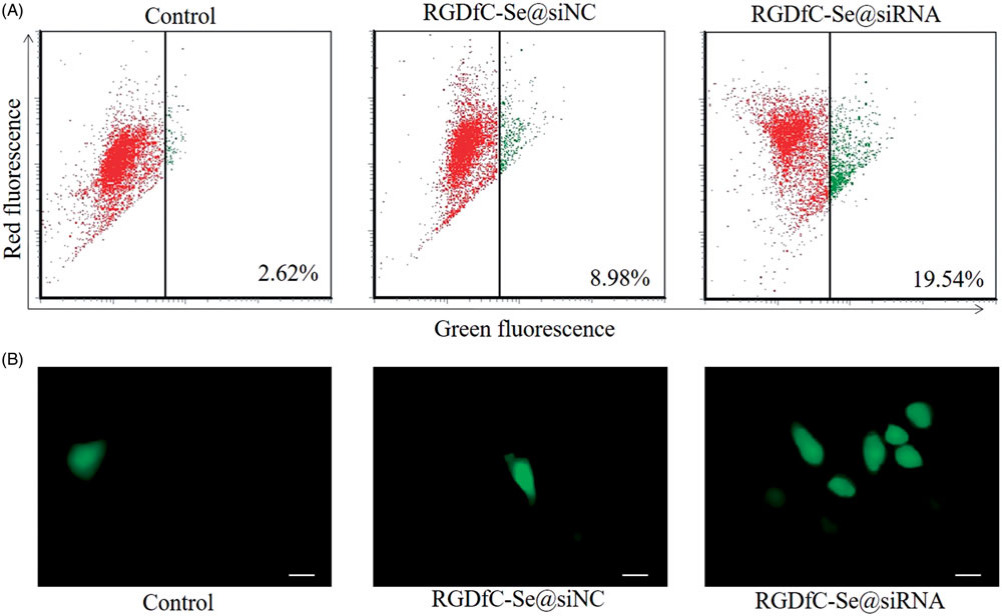
(A) Mitochondrial membrane potential (△Ψ_m_) of HeLa cells exposed to RGDfC-Se@siNC or RGDfC-Se@siRNA was examined via flow cytometry using JC-1 staining. (B) ROS overproduction induced by RGDfC-Se@siNC or RGDfC-Se@siRNA in HeLa cells was assessed via fluorescence microscope. Scale bar is 20 μm.

It has been reported that mitochondrial participation in the loss of mitochondrial membrane potential (△Ψ_m_) and the regulation of apoptosis were considered as an initial and irreversible process during apoptosis (Zhang et al., 2019). Thus, the △Ψ_m_ of cells stained with JC-1 (a mitochondria-specific dye) was examined via flow cytometry to investigate the initiation of cell apoptosis. Red and green fluorescence was observed in cells with normal polarized mitochondrial membranes and cells that lost their mitochondrial membrane potential, respectively. Figure 8(A) shows that RGDfC-Se@siRNA treatment resulted in an elevation of mitochondrial depolarization of HeLa cells, as indicated by the transformation of red fluorescence to green fluorescence. Quantitative analysis showed that the percentage of mitochondrial dysfunction in HeLa cells incubated with RGDfC-Se@siRNA increased from 2.62% (control) to 19.54%. This result indicates that RGDfC-Se@siRNA might activate the apoptotic pathway in HeLa cervical cancer cells via inducing mitochondrial dysfunction.

Chemotherapy drugs usually cause apoptosis of cancer cells via generating a large number of reactive oxygen species (ROS) (Zhan et al., 2019). Therefore, the ROS generation in HeLa cells was examined by fluorescence in this study. As shown in Figure 8(B), the treatment with RGDfC-Se@siRNA caused an obvious increase in DCF fluorescence intensity, suggesting the overproduction of ROS in HeLa cells. It was also observed that RGDfC-Se@siNC had little effect on the mitochondrial membrane potential and the intracellular ROS generation compared to the untreated cell group. These data showed that RGDfC-Se@siRNA triggered HeLa cell apoptosis possibly through mitochondrial dysfunction induced by ROS overproduction.

### *In vivo* antitumor efficacy

**Figure 9.**
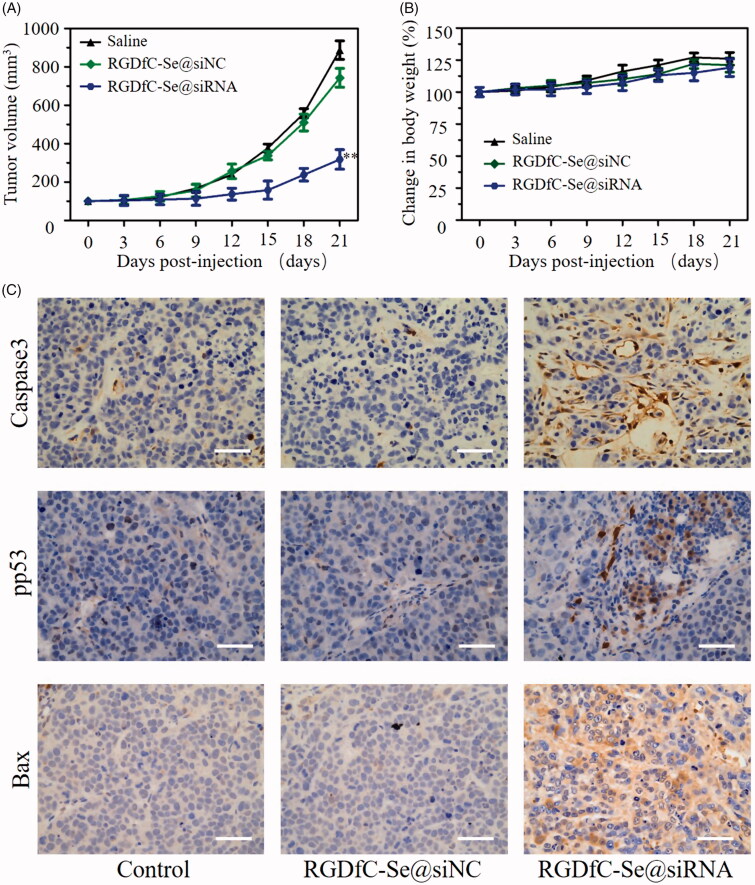
(A) The change of tumor volume after intravenous injection of saline, RGDfC-Se@siNC and RGDfC-Se@siRNA in HeLa tumor-bearing mice. **p < .01 vs. saline group. (B) The change of body weight in mice during treatment period. (C) The immunohistochemical examination of tumor tissue from mice after intravenous injection of saline, RGDfC-Se@siNC and RGDfC-Se@siRNA. Scale bar is 50 µm.

HeLa tumor xenograft was applied to confirm antitumor activity of RGDfC-Se@siRNA *in vivo*. Seen from Figure 9(A), tumor volume of the saline group rapidly increased with the increasing treatment time. However, the RGDfC-Se@siRNA treatment group exhibited much higher tumor growth delay compared with the saline control group and negative group RGDfC-Se@siNC, confirming the favorable antitumor effect of RGDfC-Se@siRNA. As shown in Figure 9(B), body weight of mouse exhibited a weak increase during the treatment time, suggesting that RGDfC-Se@siRNA did not show noteworthy toxicity *in vivo* at the dose used in this study. Histological studies were performed to further elaborate the anticancer mechanism of RGDfC-Se@siRNA *in vivo*. The apoptosis of tumor cells was investigated via pp53, caspase-3, and Bak staining (Figure 9(C)). Treatment with RGDfC-Se@siRNA observably increased the pp53-, caspase-3- and Bak-positive tumor cells compared to the saline or RGDfC-Se@siNC-treatment group, suggesting that RGDfC-Se@siRNA could clearly induce the apoptosis of tumor cells. These results showed that RGDfC-Se@siRNA presents great potential in cervical cancer treatment via inducing the apoptosis of cervical cancer cells.

### *In vivo* toxicity assessment

**Figure 10.**
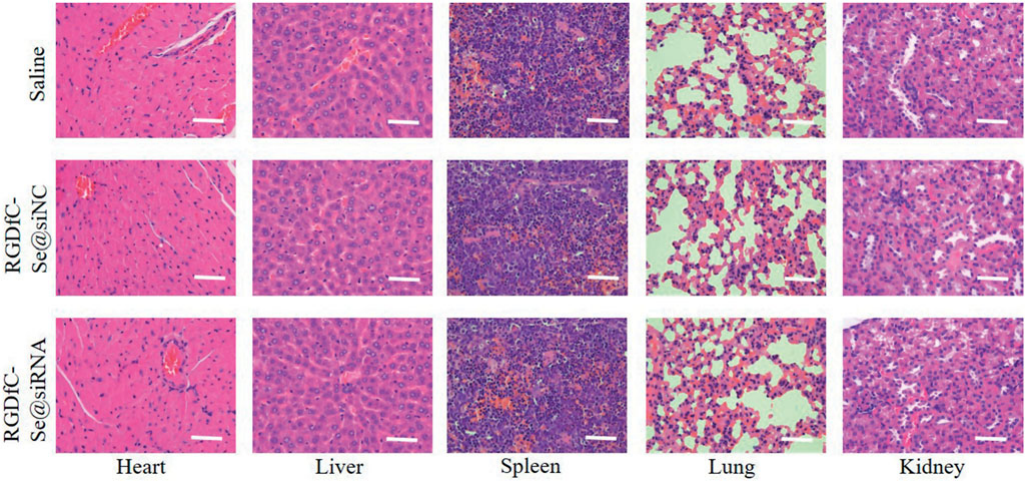
H&E analyses of heart, liver, spleen, lung, and kidney from mice after intravenous injection of saline, RGDfC-Se@siNC and RGDfC-Se@siRNA. Scale bar is 50 µm.

In order to examine the potential toxicity of RGDfC-Se@siRNA *in vivo*, the heart, liver, spleen, lung, and kidney of mice was studied via H&E staining. Seen from Figure 10, no apparent abnormalities in any of the five organs were observed in RGDfC-Se@siRNA group compared with saline control or negative RGDfC-Se@siNC groups. This result confirmed that RGDfC-Se@siRNA possesses satisfactory biocompatibility *in vivo*. Therefore, RGDfC-Se@siRNA exhibited the potential to be a promising nanoscale tumor-targeting anticancer drug candidate for cervical cancer therapy.

## Conclusions

In the current research, tumor-targeting nanoparticles (RGDfC-Se@siRNA) were prepared to deliver Derlin1-siRNA to HeLa cells for human cervical cancer treatment. HeLa cells exhibited significant uptakes of RGDfC-Se@siRNA via clathrin-associated endocytosis in HeLa cells and showed quicker release of siRNA from RGDfC-SeNPs under acidic conditions. RGDfC-Se@siRNA effectively silenced the Derlin1 gene in HeLa cells and suppressed the proliferation and invasion/migration of HeLa cells. RGDfC-Se@siRNA triggered apoptosis of HeLa cells through the ROS-mediated mitochondrial pathway. Furthermore, RGDfC-Se@siRNA was highly efficient in inhibiting the growth of cervical cancer *in vivo*. Taken together, the current research provides a promising strategy for designing siRNA carriers for the treatment of cervical cancer.
